# A Novel Approach for Microbial Synthesis of Enantiomerically Pure Whisky Lactones Based on Solid-State Fermentation

**DOI:** 10.3390/molecules23030659

**Published:** 2018-03-14

**Authors:** Filip Boratyński, Ewa Szczepańska, Aleksandra Grudniewska, Bartłomiej Skalny, Teresa Olejniczak

**Affiliations:** Department of Chemistry, Wroclaw University of Environmental and Life Sciences, 50-375 Wrocław, Poland; ewa.szczepanska@upwr.edu.pl (E.S.); aleksandra.grudniewska@upwr.edu.pl (A.G.); bartlomiej.skalny@wp.pl (B.S.); teresa.olejniczak@upwr.edu.pl (T.O.)

**Keywords:** whisky lactone, aroma compounds, kinetic resolution, solid-state fermentation, agroindustrial side stream, rapeseed cake

## Abstract

In this study, solid-state fermentation (SSF) was proposed as an alternative approach to obtain optically pure forms of one of the most common aroma compounds, whisky lactone. Filamentous fungi were used for enantioselective hydrolysis of a racemate of *trans* and *cis* whisky lactones, utilizing rapeseed cake as a growth medium. Among the tested fungi, *Fusarium oxysporum* AM13 and *Papularia rosea* AM17 were chosen for further studies. Various process parameters, including temperature, moisture content of solid media, and substrate concentration were optimized to maximize the efficiency of the kinetic resolution process. After optimization of the culture conditions (33 °C temperature, 60% moisture content, and substrate concentration of 3 mg/g oilseed cake), *F. oxysporum* AM13 resolved a mixture of *trans*-(+)-(4*S*,5*R*) and *cis*-(+)-(4*R*,5*R*) whisky lactones with enantiomeric excess (ee), ee > 99% and ee = 98%, respectively. This study presents an inexpensive and environmentally friendly method for the production of enantiomerically pure aroma lactones via the solid-state fermentation of oilseed cake. The results revealed that SSF is an effective method for acquiring highly valued and industrially demanded compounds with negligible economic cost.

## 1. Introduction

Lactones are highly valued aroma compounds used in the preparation of various foodstuffs. One of the commonly known and industrially used fragrances is whisky lactone. This compound, which uses oak wood, is also known as oak or quercus lactone. It is found in aged alcoholic beverages, such as whisky, cognac, brandy, and wine [1,2]. Each of the four stereoisomers of whisky lactone provides a different fragrance: *cis* isomers are described as having an earthy and woody fragrance, while *trans* isomers are reminiscent of celery [3]. Individual *trans* and *cis* isomers occur in nature; mainly, oak wood *trans*-(+)-(4*S*,5*R*) and *cis*-(−)-(4*S*,5*S*) isomers have been determined [4,5]. However, commercially available whisky lactone, which is used as an additive in the food industry, contains a racemate of *trans* and *cis* isomers. There is need to perform a systematic study on the relationship between whisky lactone’s structure and its other biological activity. Therefore, the development of profitable biotechnological methods to resolve individual stereoisomers of whisky lactone is necessary.

Methods based on chemical asymmetric synthesis are well described in the literature. One approach to whisky lactone synthesis is the regiocontrolled alkylation of 2-(trimethylsiloxy)-furan [6]. An asymmetric Michael reaction of this compound was also applied to obtain *trans*-(4*S*,5*R*)-isomer [7]. Jiang et al. [8] synthesized (−) and (+)-*trans* whisky lactones by the iodolactonization of propargyl alcohol. Enantiomerically pure *cis* and *trans* isomers were obtained from acyclic vinyl sulfoxides [9]. Enantioselective dialkyl zinc addition to aldehydes resolved (4*S*,5*S*)-isomer with ee = 94% [10]. 

Notably, the current chemical asymmetric syntheses require the use of environmentally harmful organic solvents, such as toluene, cyclohexane, tetrahydrofuran, or heavy metals. Conversely, the biotransformation approach enables the synthesis of whisky lactone under milder conditions, which are suitable for microorganisms and the activity of their enzymes. However, compounds obtained from processes regarded as natural, such as biotransformation, are increasingly gaining attention [11]. In the literature, there are only a few known biotechnological methods to obtain enantiomerically-enriched whisky lactone. One applies alcohol dehydrogenases as biocatalysts to enantioselective oxidation of racemic *threo*- and *erythro*-3-methyloctane-1,4-diols [12]. Another uses microbial lactonization of γ-oxoacids [12]. The application of lipases in a multi-step chemoenzymatic synthesis of optically active whisky lactones was also described [13]. 

Presently, microbial solid-state fermentation (SSF) using renewable food waste is an excellent method for the efficient production of industrially important hydrolases [14,15,16,17,18,19,20,21,22,23]. Moreover, SSF can constitute a more profitable alternative to the microbial processes conducted by submerged fermentation (SmF). Literature data has claimed that product yields are higher in SSF systems than in SmF systems [24]. SSF systems simulate the living conditions of filamentous fungi and are widely used in the food industry. Due to the preference of fungi for low water activity, only a minimum water consumption is required and the probability of yeast and bacteria contamination is negligible. Therefore, there is low effluent in water production and no necessity to use antifoam agents. Moreover, the high-energy stirring process in SmF can be eliminated in SSF [25]. Additional economic benefits of SSF in comparison to SmF processes result from the application of low-cost agroindustrial wastes (wheat bran, fruit pomace, vegetable bagasse, oilseeds cake) [19,26,27,28]. Fine chemicals, such as enantiopure forms of chiral compounds, can be manufactured using agricultural by-products [29].

Oilseed cake is a solid residue obtained from the vegetable oil production process and constitutes up to 75% of the seed weight. Owing to the content of nutrient compounds, such as carbohydrates, fat, and proteins, it can be used as a wholesome medium for the growth of microorganisms [20,30]. 

Currently, the application of biocatalytic kinetic resolution in the field of fragrances—such as menthol, methyl jasmonate, ionones, and damascones—is well known only in SmF systems [31]. Besides our preliminary results indicating the potential application of agroindustrial by-products to chiral compound production [32], no other studies have described the separation of high-value individual enantiomers of whisky lactones by microbial enantioselective hydrolysis of its racemate. Due to its novelty, joining two SSF processes with subsequent biotransformation, the method still requires continuous improvement.

The aim of this study was to show the possibility of using low-cost agroindustrial residues as a growth medium for microorganisms, conducted in a kinetic resolution of whisky lactone, rendering them as enantiomerically pure forms. Using rapeseed cake, various filamentous fungi were studied for their ability to enantioselectively hydrolyze a diastereoisomeric mixture of whisky lactone. To increase the efficiency of the production of pure enantiomers, cultivation parameters were optimized for the chosen fungus. Considering the wide range of applications of whisky lactones in the food industry as well as the economic benefits owing to the sustainable management of agricultural by-products, this approach should gain increasing attention.

## 2. Results

**Figure 1:**
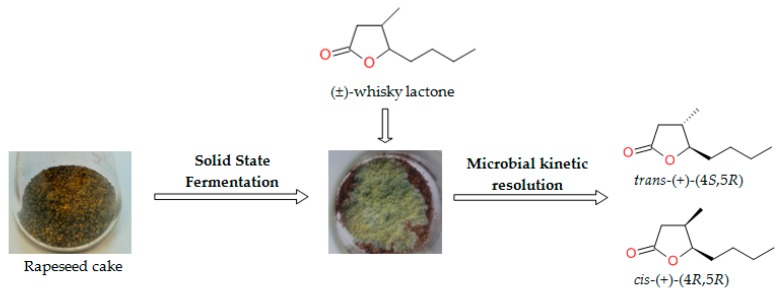
Microbial kinetic resolution of whisky lactone by solid-state fermentation (SSF).

As a result of the kinetic resolution of a racemate of *trans* and *cis* whisky lactones (*trans*/*cis* ratio: 50%/50%) conducted by filamentous fungi and utilizing rapeseed cake as a biotransformation medium, two enantiomerically-enriched isomers of whisky lactones, *trans*-(+)-(4*S*,5*R*) and *cis*-(+)-(4*R*,5*R*), were obtained. Solid-state fermentation followed by the biotransformation of the diastereoisomeric mixture of whisky lactone is shown in Figure 1. 

Selected filamentous fungi exhibited the biocatalytic ability to hydrolyze the internal ester bond in whisky lactones (Table 1). The biotransformation process was controlled after 3, 6, and 10 days; however, after 10 days of cultivation, an increase in the enantiomeric excess (ee) of whisky lactone was not observed. Additionally, we determined the diastereoisomeric excess of *trans* isomers relative to *cis* isomers (*trans*/*cis* ratio). Initially, the predominance of *trans* diastereoisomer of whisky lactone in the culture of *Aspergillus* sp. AM31, *Penicillum camemberti* AM83, *P. chrysogenum* AM112, and *P. notatum* AM904 was observed. Subsequently, *trans*-whisky lactone was hydrolyzed and diastereoisomeric excess of *trans* isomers decreased. During biotransformation catalyzed by *Pycnidiella resinae* AM50, the *cis* diastereoisomer was noted to be dominant. In the case of *Fusarium culmorum* AM9, *F. equiseti* AM15, *F. oxysporum* AM13, and *Papularia rosea* AM17, the *trans*/*cis* ratio was stable. *F. culmorum* AM9, *F. equiseti* AM15, and *P. camembertii* AM83 performed stereoselective hydrolysis within three days; thereafter, the process remained unchanged. Notably, *F. equiseti* AM15 proceeded transformation with opposite stereoselectivity, and *trans* (−)-(4*R*,5*S*) isomer was obtained. *P. vermiculatum* AM30 hydrolyzed whisky lactones completely, indicating a high efficiency of hydrolase biosynthesis during SSF. 

The enantiomeric excess of whisky lactones in the biotransformation catalyzed by *Aspergillus* sp. AM31, *F. oxysporum* AM13, *P. rosea* AM17, and *P. notatum* AM904 increased with time. In the transformation of *F. oxysporum* AM13 and *P. rosea* AM17 (+)-(4*S*,5*R*)-isomer with ee = 56 and 70%, and (+)-(4*R*,5*R*)-isomer with ee = 60 and 42%, respectively were obtained. Therefore, two promising strains, *F. oxysporum* AM13 and *P. rosea* AM17 (Table 1), were selected for further optimization of the culture conditions. Initial optimization of hydrolysis involved a modification of the temperature and moisture content. The impact of these two factors on the kinetic resolution is presented in Table 2.

Filamentous fungi catalyzed kinetic resolution within six days and further progress was not observed. Considering the screening results, the progress of biotransformation was monitored after two and five days. In the case of *F. oxysporum* AM13, independent of temperature, the increase in rapeseed cake moisture from 40 to 60% caused a rise in the enantiomeric purity of both isomers of whisky lactones. At 100% moisture, the efficiency of the biotransformation was the lowest. The optimal conditions were 33 °C temperature and 60% oilseed cake moisture. *F. oxysporum* AM13 afforded (+)-(4*S*,5*R*)-isomer with ee = 98% and (+)-(4*R*,5*R*)-isomer with ee = 82% within five days. For *P. rosea* AM17, an analogous change in conditions did not provide the expected results. At a higher temperature, *P. rosea* AM17 completely decomposed both isomers of whisky lactones. Presumably, stressful conditions caused intensive biosynthesis of non-selective hydrolases.

**Figure 2:**
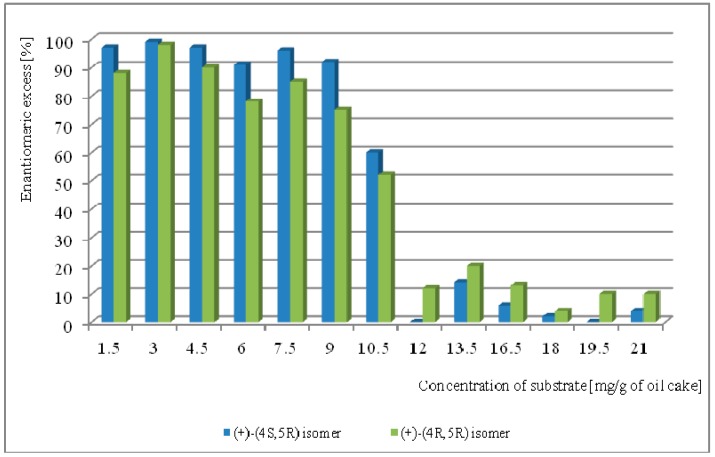
Effect of substrate concentration on biotransformation of diastereoisomeric mixture of whisky lactones catalyzed by *F. oxysporum* AM13 after five days (in % according to GC).

The concentration of whisky lactones to be added to the culture was the next parameter to be optimized. Based on previous optimization studies, *F. oxysporum* AM13 was selected. The impact of substrate concentration on the hydrolysis process is shown in Figure 2.

The cultures were conducted using previously determined optimal conditions (33 °C temperature, 60% moisture). Biotransformation in the *F. oxysporum* AM13 culture with a whisky lactone concentration ranging from 1.5 to 9 mg/g of rapeseed cake afforded whisky lactones with high enantiomeric excess. Most importantly, the addition of whisky lactones at a concentration of 3 mg/g oilseed cake yielded an enantiomerically pure form of (+)-(4*S*,5*R*)-isomer (ee > 99%) and high ee of (+)-(4*R*,5*R*)-isomer (ee = 98%). Whisky lactones at an amount higher than 10.5 mg/g oilseed cake inhibited fungal growth and the enantiomeric excess of the whisky lactones rapidly decreased. The impact of the initial and final biotransformation conditions on the whisky lactone kinetic resolution process using *F. oxysporum* AM13 as a biocatalyst is shown in Table 3.

**Figure 3:**
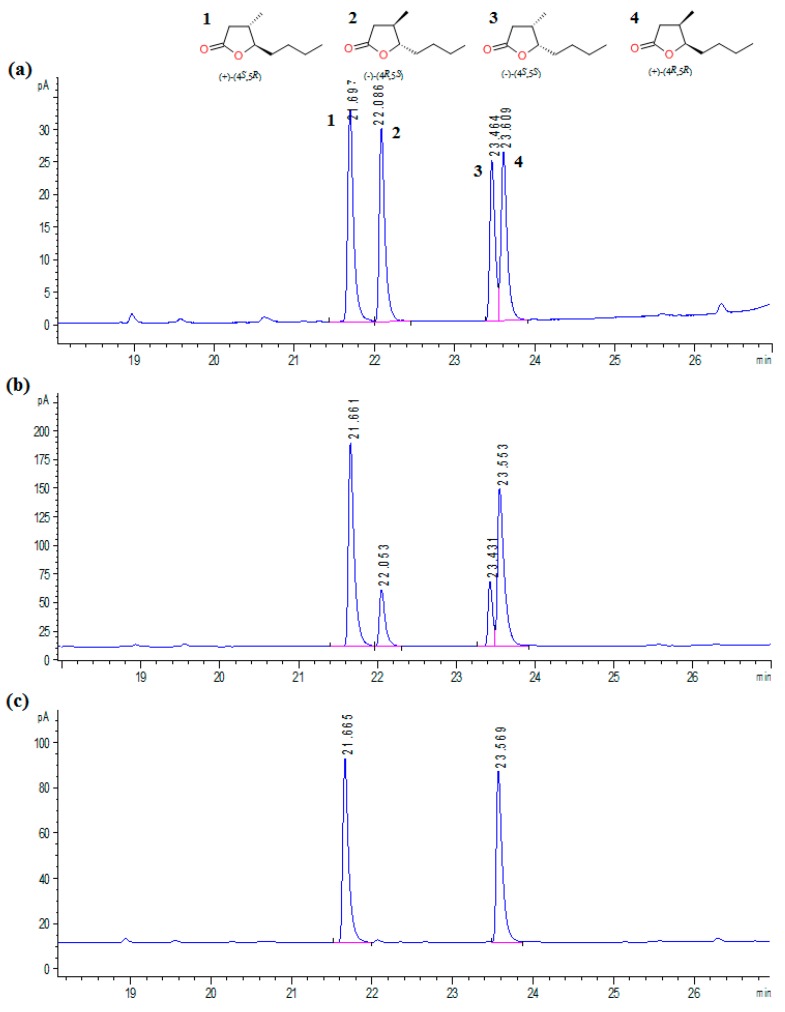
Chromatograms of substrate (racemate *trans* and *cis* whisky lactones) used for microbial kinetic resolution (**a**) and enantiomerically-enriched isomers of whisky lactones obtained before (**b**) and after the optimization process (**c**).

Chromatographic results for whisky lactone isomers obtained before and after the optimization of the microbial hydrolysis process in comparison to an initial diastereoisomeric mixture of *trans* and *cis* whisky lactones are presented in Figure 3.

In our recently published study, we showed for the first time the possibility of using oilseed cakes, including rapeseed and linseed cake, for the production of different optically-active aroma compounds, including whisky lactone. However, whisky lactone was obtained using *F. oxysporum* AM13 with modest enantiomeric excess (ee = 56% for *trans*-(4*S*,5*R*)-isomer and ee = 60% for *cis*-(4*R*,5*R*)-isomer). Therefore, we decided to optimize the biotransformation conditions to significantly increase the enantiomeric excess of both isomers. Apart from this novel approach to biotransformation conducted on solid-state medium described above, only two biotechnological methods for the synthesis of whisky lactone isomers have been proposed until now. In the first method, enantioselective oxidation of racemic *threo*- and *erythro*-3-methyloctane-1,4-diols was conducted using alcohol dehydrogenase isolated from horse liver (HLADH), recombinant from *Escherichia coli* (rec-HLADH), and primary alcohol dehydrogenase (PADH II) as biocatalysts. Thereafter, (−)-(4*R*,5*S*) and (+)-(4*R*,5*R*) isomers with ee = 27–82% were formed [12]. Additional experiments included the microbial lactonization of γ-oxoacids. The lactonization of 3-methyl-4-oxooctanoic acid catalyzed by *Beauveria bassiana* AM278 derived a mixture of *trans* (+)-(4*S*,5*R*) and *cis* (−)-(4*S*,5*S*) whisky lactone isomers with ee > 99% and ee = 77%, respectively. As a result of the same process, *trans*-(+)-(4*S*,5*R*) isomer was produced by *Didimospheria igniaria* KCH6651, *Laetiporus sulphurens* AM525, *Chaetomium* sp. KCH6670, and *Saccharomyces cerevisiae* AM464 with ee 99% [12]. It is worth mentioning that both methods that we developed were conducted using commercial enzymes or during the SmF process, which required higher capital investments for particular bioprocesses in comparison to SSF. During our study, the application of agroindustrial by-products to straightforward biotransformation processes allowed us to obtain comparable or even higher enantiomeric purity of whisky lactones in comparison to the biotechnological methods tested so far. 

## 3. Materials and Methods 

### 3.1. Materials

Rapeseed cake was purchased from Oleofarm, Wroclaw, Poland. Mixture of *trans* and *cis* whisky lactones (5-butyl-4-methyldihydro-2(3*H*)-furanone) was purchased from Sigma-Aldrich Chemical Co. (St. Louis, MO, USA). Microorganisms used for screening (*Aspergillus* sp. AM31, *Fusarium culmorum* AM9, *Fusarium equiseti* AM15, *Fusarium oxysporum* AM13, *Papularia rosea* AM17, *Penicillum camembertii* AM83, *Penicillium chrysogenum* AM112, *Penicillium notatum* AM904, *Penicillium vermiculatum* AM30, *Pycnidiella resinae* AR50) were obtained from the Department of Chemistry, Wroclaw University of Environmental and Life Sciences (Poland). They were stored at 4 °C on Sabouraud agar slants containing peptone (10 g), glucose (30 g), and agar (15 g) dissolved in water (1 L) at pH 5.5.

### 3.2. Solid-State Fermentation

Rapeseed cake was placed (5 g each) in Erlenmayer flasks and autoclaved for 15 min at 121 °C. It was then hydrated to 60% moisture, inoculated with 0.5 mL of a dense spore suspension 2.3 × 10^7^ spores/mL prepared in sterile water from agar slant cultures, and thoroughly mixed. Flasks were incubated in thermostatic cabinet at 30 °C with defined humidity and without shaking.

### 3.3. Biotransformation Process

After three days of cultivation, grown cultures were sprayed with a 0.2 mL of 5 mM solution of substrates in acetone and water (1:1 *v*/*v*). For each biotransformation, three individual flasks were set up to monitor the progress of reaction after 3, 6, and 10 days. After each period, distilled water (15 mL) and ethyl acetate (5 mL) were added for extraction. Media were vortexed for five minutes at 3500 rpm and centrifuged at 5000 rpm for 15 min at room temperature. Finally, the organic phase was dehydrated by anhydrous MgSO_4_ and transferred to a vial. Each sample was then analyzed on a gas chromatography instrument equipped with an autosampler. In control experiments, whisky lactones were incubated in sterile rapeseed cakes without a bioacatalyst to check substrate stability. Additionally, to estimate the fungal metabolites, control cultures were performed without addition of substrate.

### 3.4. Optimization Process

The effect of temperature incubation on hydrolysis was assessed in 23, 28 and 33 °C. Moisture content was tested at 40, 60, 80, and 100% according to Equation (1):

(1) $$M_{gm}=\frac{m_{w}}{m_{w}+m_{rc}}\times 100\%,$$

where *M_gm_* is the moisture in the growth medium, *m_w_* is mass of water added to rapeseed cake and *m_rc_* means mass of used rapeseed cake to the individual SSF. 

The impact of the initial substrate concentration was evaluated by increasing the amount of added substrate from 1.5 to 20 mg/g of rapeseed cake. Biotransformation progress was monitored after two and five days.

### 3.5. Analysis

The progress of reaction and enantiomeric excesses of the hydrolysis products was determined by gas chromatography. Determination of the individual isomers was based on previously obtained standards of chiral lactones [12]. Quantification was made by comparing with standard graphs drawn for individual compounds. Gas chromatography analysis (FID, carrier gas H_2_) was carried out on Agilent Technologies 7890N (GC System, Santa Clara, CA, USA). Enantiomeric excesses of the products were determined on chiral column Cyclosil-B (30 m × 0.25 mm × 0.25 μm) according the next temperature program: 80 °C, 160 °C (3 °C/min), 250 °C (20 °C/min) (3 min). Samples (2 μL) were injected with split 9:1; the flow of carrying gas was 1 mL/min. The total run time was 34.0 min. Retention times were established as follow: (+)-(4*S*,5*R*) = 21.66 min, (–)-(4*R*,5*S*) = 22.01 min, (–)-(4*S*,5*S*) = 23.45 min, (+)-(4*R*,5*R*) = 23.60 min. The structures of the compounds were confirmed on the basis of ^1^H NMR and ^13^C NMR, which were recorded for CDCl_3_ solutions on a Bruker Avance DRX 600 (600 MHz) spectrometer (Billerica, MA, USA).

## 4. Conclusions

In summary, the results of this study demonstrated the possibility of microbial kinetic resolution of whisky lactones as an approach to obtain enantiomerically pure *trans* and *cis* isomers. The application of renewable agricultural by-products, i.e., rapeseed cake, as a growth medium is an especially novel approach. The established optimal conditions (33 °C temperature, 60% moisture content, and substrate concentration of 3 mg/g oilseed cake) using *F. oxysporum* AM13 resolved *trans*-(+)-(4*S*,5*R*) and *cis*-(+)-(4*R*,5*R*)-isomers of whisky lactone with ee > 99% and ee = 98%, respectively. This study presented an inexpensive and environmentally friendly method for the production of enantiomerically pure aroma lactones via the solid-state fermentation of oilseed cake. The proposed biotechnological method is a solution for two issues: increased demand for food additives and the sustainable management of food waste.

## Figures and Tables

**Table 1:** Composition of isomers of whisky lactone after microbial kinetic resolution of diastereoisomeric mixture of whisky lactone (in % according to GC).

Strain	Time (Days)	*trans*/*cis* Ratio (%)	*trans*-(+)-(4*S*,5*R*) ee ^a^ (%)	*cis*-(+)-(4*R*,5*R*) ee ^a^ (%)
*Aspergillus* sp. AM31	3	67/33	0	0
	6	59/41	38	28
*Fusarium culmorum* AM9	3	48/52	12	14
	6	55/45	16	12
*Fusarium equiseti* AM15	3	50/50	24 ^b^	18
	6	50/50	24 ^b^	18
*Fusarium oxysporum* AM13	3	56/44	0	0
	6	56/44	56	60
*Papularia rosea* AM17	3	38/62	46	26
	6	33/67	70	42
*Penicillum camemberti* AM83	3	77/23	32	0
	6	57/43	30	10
*Penicillium chrysogenum* AM112	3	77/23	24	20
	6	53/47	42	14
*Penicillium notatum* AM904	3	70/30	0	0
	6	58/42	52	12
*Penicillium vermiculatum* AM30	3	-/-	-	-
	6	-/-	-	-
*Pycnidiella resinae* AM50	3	41/59	23	0
	6	27/73	40	0

^a^ ee—enantiomeric excess; ^b^ The biotransformation proceeded with opposite enantiomer selectivity ((−)-(4*R*,5*S*)).

**Table 2:** Effect of temperature and moisture content on biotransformation of diastereoisomeric mixture of whisky lactone (in % according to GC).

Strain	Temperature (°C)	Moisture (%)	2 Days			5 Days		
			*trans*/*cis* Ratio (%)	*trans*-(+)-(4*S*,5*R*) ee ^a^ (%)	*cis*-(+)-(4*R*,5*R*) ee ^a^ (%)	*trans*/*cis* Ratio (%)	*trans*-(+)-(4*S*,5*R*) ee ^a^ (%)	*cis*-(+)-(4*R*,5*R*) ee ^a^ (%)
*Fusarium oxysporum* AM13	23	40	53/47	28	24	56/44	68	50
		60	55/45	58	40	61/39	88	64
		80	56/44	68	38	59/41	88	54
		100	56/44	56	38	60/40	90	60
	28	40	56/44	56	42	62/38	84	76
		60	64/36	82	44	62/38	76	38
		80	70/30	24	16	68/32	38	16
		100	71/29	18	12	69/31	20	18
	33	40	60/40	48	44	60/40	58	66
		60	66/34	70	66	61/39	98	82
		80	63/37	72	60	68/32	88	74
		100	62/38	44	40	65/35	78	54
*Papularia rosea* AM17	23	40	51/49	30	26	34/66	78	36
		60	60/40	26	14	62/38	52	24
		80	73/27	10	10	70/30	18	16
		100	73/27	8	14	69/31	22	16
	28	40	40/60	40	26	38/62	52	30
		60	43/57	86	32	38/62	86	34
		80	57/43	36	28	54/46	52	34
		100	68/32	8	6	-	-	-
	33	40	35/65	72	30	-	-	-
		60	53/47	60	10	-	-	-
		80	51/49	56	16	-	-	-
		100	59/41	40	14	-	-	-

^a^ ee—enantiomeric excess.

**Table 3:** The comparison of the enantiomeric purity of whisky lactone isomers obtained before and after optimization of the biotransformation process.

Conditions	Parameters			Enantiomeric Excess	
	Temperature (°C)	Moisture (%)	Concentration of Substrate (mg/g of Oil Cake)	*Trans*-(+)-(4*S*,5*R*) (%)	*Cis*-(+)-(4*R*,5*R*) (%)
Initial	23	60	1.5	56	60
Optimized	33	60	3	> 99	98
